# Unilateral delayed post-hypoxic leukoencephalopathy: a case report

**DOI:** 10.1186/s13256-022-03701-3

**Published:** 2022-12-26

**Authors:** Johannes A. R. Pfaff, Lukas Machegger, Eugen Trinka, Johannes Sebastian Mutzenbach

**Affiliations:** 1 Department of Neuroradiology, Christian Doppler Medical Center, Paracelsus Medical University, Ignaz-Harrer-Straße 79, 5020 Salzburg, Austria; 2 Department of Neurology, Centre for Cognitive Neuroscience, Christian Doppler Medical Center, Paracelsus Medical University, Salzburg, Austria; 3 Neuroscience Institute, Centre for Cognitive Neuroscience, Christian Doppler Medical Center, Paracelsus Medical University, Salzburg, Austria; 4 Karl Landsteiner Institute for Neurorehabilitation and Space Neurology, Salzburg, Austria; 5 Spinal Cord Injury and Tissue Regeneration Centre, Salzburg, Austria

**Keywords:** Ischemic stroke, Carotid artery, internal, dissection, Hypoxia-ischemia, brain, Leukoencephalopathies, Case reports

## Abstract

**Background:**

Delayed post-hypoxic leukoencephalopathy is a rare entity following hypoxia. Clinical and radiological signs of delayed post-hypoxic leukoencephalopathy have not previously been reported following acute ischemic stroke.

**Case presentation:**

We report a case of an 81-year-old Central European man who presented with a dissection-related occlusion of the left carotid artery. He showed clinical improvement immediately after endovascular stroke therapy, followed by a significant clinical and especially cognitive deterioration thereafter and a clinical recovery after several weeks. The clinical course of the patient was accompanied by morphological changes on magnetic resonance imaging characteristic of delayed post-hypoxic leukoencephalopathy; that is, strictly limited and localized unilaterally to the left anterior circulation.

**Conclusion:**

This case demonstrates that clinical symptoms and morphological changes on magnetic resonance imaging compatible with delayed post-hypoxic leukoencephalopathy do not necessarily only occur with global hypoxia, but can also occur in patients with a large vessel occlusion in the corresponding vascular territories.

## Highlights

- Clinical symptoms suggestive of delayed post-hypoxic leukoencephalopathy (DPHL) can be observed in very rare cases following successful revascularization therapy in large vessel occlusions, causing acute ischemic stroke.
- Radiological signs of DPHL may be subtle, can be unilateral, and therefore can be easily overlooked.
- Repeated controls using magnetic resonance imaging (MRI) is necessary to correlate and monitor the clinical course and the morphological changes of the brain over time.

## Background

The brain is dependent on a continuous supply of blood, which, among other things, transports oxygen and glucose for metabolism. Disruption of cerebral blood flow very rapidly results in a hypoglycemic, hypoxic state that leads to cell death. [1] In the event of a failure of the cardiovascular system, for example, cardiac arrest, blood supply to the whole brain and thus the supply of oxygen is interrupted. Therefore, despite successful resuscitation measures, patients can develop delayed post-hypoxic leukoencephalopathy (DPHL) [2]. DPHL is a rare entity clinically characterized by neurologic deterioration following a period of clinical stability or improvement after an initial episode of hypoxia [3].

Unlike cardiovascular failure, which causes global hypoxia, an ischemic stroke only deprives a part of the brain of adequate oxygen supply. Recanalization therapies, such as intravenous thrombolysis or endovascular stroke treatment, aim to restore cerebral blood flow and resolve this hypoxic state.

Our report entails a patient who, following an occlusion of the left cervical internal carotid artery, shows both the clinical course and characteristic image-morphological changes for DPHL; that is, strictly limited and localized unilaterally to the left anterior circulation. We provide a detailed description of the patient’s course of treatment as well as clinical and radiological findings in light of this extremely rare constellation.

## Case presentation

An 81-year-old Central European man was hospitalized in a tertiary hematology and oncology center for diagnostic work-up and treatment of acute myeloid leukemia (AML). Past medical history revealed a chronic kidney disease (G2), arterial hypertension, hyperlipidemia, benign prostatic hyperplasia, and hepatic steatosis. The patient was married and had no history of smoking, drugs, or alcohol abuse. The patient’s height was 1.80 m, his weight was 90 kg, and his body mass index (BMI) was 27.8 kg/m^2^. The patient worked as a farmer and was retired for more than 15 years. Due to the high age of the patient, family history could only be collected to a limited extent. As far as can be ascertained, the family history did not reveal any risk factors or cancer.

On day 13 after initial admission, the patient suddenly developed right-sided hemiparesis and global aphasia [National Institutes of Health Stroke Scale (NIHSS) 17, Modified Rankin Scale (mRS) 5]. Initial imaging showed no signs of early ischemic changes on non-contrast-enhanced computed tomography of the brain (NCCT; Alberta Stroke Program Early CT score 10) and an occlusion of the left cervical carotid artery, but no intracranial large vessel occlusion on CT-angiography (images not shown). Due to the sudden onset of symptoms and the severity of the symptoms, the occlusion of the left internal carotid artery was classified as acute, and the patient was transferred to our comprehensive stroke center for endovascular treatment. Upon arrival at the comprehensive stroke center, the patient was conscious with vital signs including temperature of 36.6 °C, heart rate of 100 beats per minute (bpm), blood pressure of 150/93 mmHg, and oxygen saturation of 99%. Electrocardiogram (ECG) showed a sinus rhythm without any signs of myocardial ischemia. Relevant laboratory values are listed in Table 1.

**Table 1:** Laboratory values with a reference to the relevant events

	Unit	Day of endovascular stroke treatment (EST)	Day 10 after EST = MRI 1	Day 23 after EST = MRI 2	Day 92 after EST = MRI 3
RBC	T/L	2.8	2.9	3.8	3.7
HGB	g/dl	8.6	8.8	11.9	11.9
HCT	%	24.5	24.5	35.3	33.6
WBC	G/L	21.39	1.59	4.57	2.11
PLT	G/L	29	40	343	156
SGOT	U/l	53	25	16	26
SGPT	U/l	28	17	10	11
Urea	mg/dl	79	41	38	27
Cr	mg/dl	0.88	0.46	0.73	0.57
Glucose	mg/dl	101	89	141	79
INR		1.1	1.2	1.1	
CRP	mg/dl	6.2		1.1	
Na	mmol/l	143	140	137	141
K	mmol/l	3.7	3.5	3.6	3.5
Cl	mmol/l	106	106	103.4	108
Ca	mmol/l	2.16	2.09	2.16	2.23

*Ca*  calcium, *Cl*  chloride, *Cr*  creatinine, *CRP*  C-reactive protein, *EST*  endovascular stroke treatment, *HGB*  hemoglobin, *HCT*  hematocrit, *INR*  international normalized ratio, *K*  potassium, *MRI*  magnetic resonance imaging, *Na*  sodium, *PLT*  platelets, *RBC*  red blood cells, *SGOT*  serum glutamic oxaloacetic transaminase, *SGPT*  serum glutamic pyruvic transaminase, *WBC*  white blood cells

To treat the occlusion of the left internal carotid artery, endovascular therapy under general anesthesia was initiated. Catheter angiography confirmed the cervical occlusion of the left internal carotid artery caused by a limited subpetrous dissection. Mechanical recanalization was performed using catheterization with aspiration only. Owing to thrombocytopenia with a platelet count of 29 G/L, secondary to AML, neither a stent was implanted nor systemic thrombolysis administered as bridging. Final angiographic runs showed no evidence of a cervical or intracranial vascular occlusion (corresponding to a reperfusion according to the extended Thrombolysis in Cerebral Infarction grading, eTICI 3). Onset to recanalization time was 265 minutes.

**Fig. 1:**
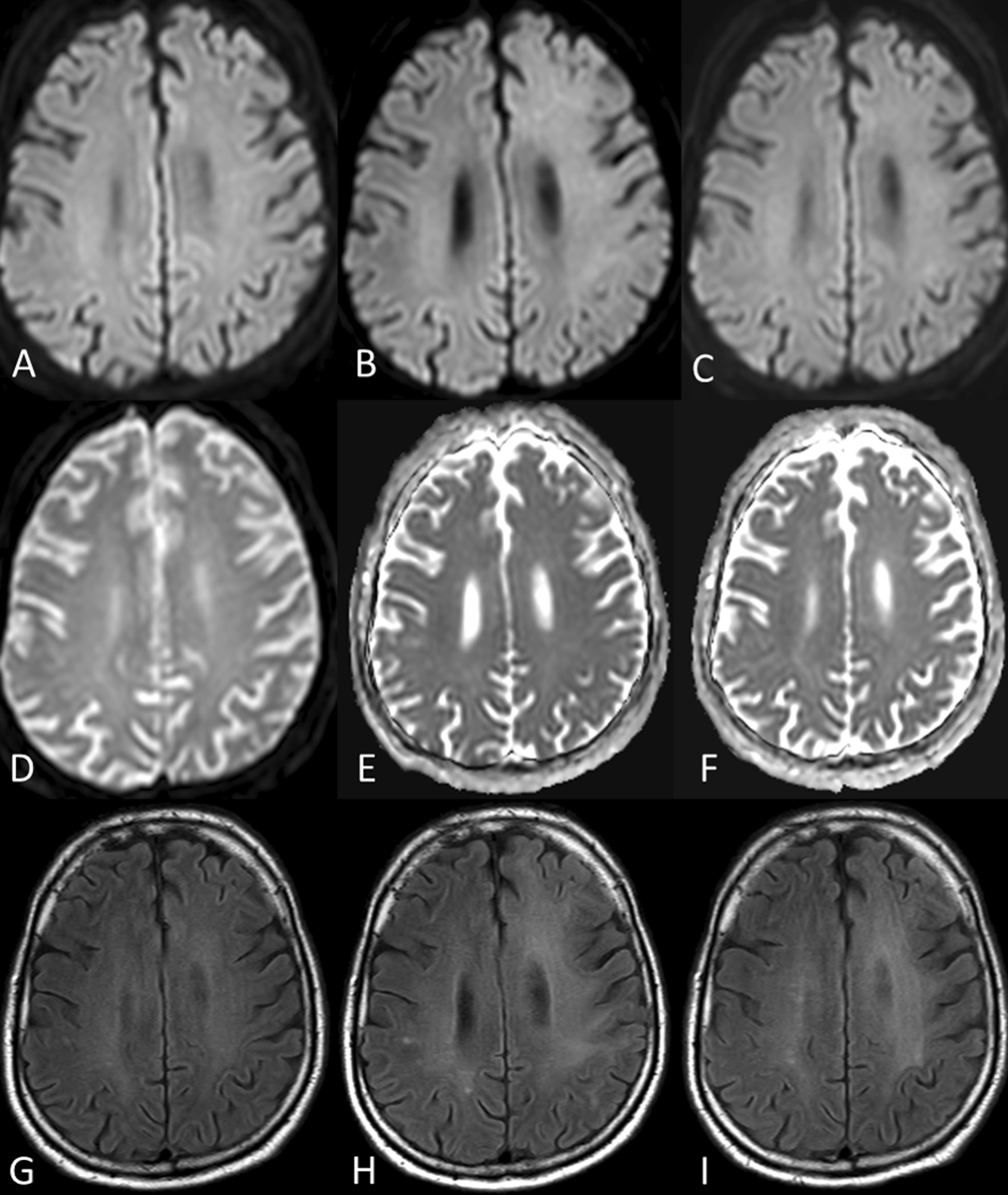
Diffusion-weighted imaging (DWI, top row), apparent diffusion coefficient (**A**, **D**, **C**, middle row) and fluid attenuated inversion recovery (FLAIR, bottom row) imaging at day 10 (**A**, **D** and **G**), day 23 (**B**, **E** and **H**), and day 92 (**C**, **F** and **I**) after endovascular recanalization of a left internal carotid artery occlusion. Please note the homogeneous white matter hyperintensity in the left anterior and middle cerebral artery territory on FLAIR images, which is not visible on day 10 (**G**), but on day 23 (**H**) and also on day 92 (**I**), sparing the subcortical U-fibers and with inconspicuous DWI at each corresponding MRI

The patient showed clinical improvement in aphasia and hemiparesis immediately after extubation. NCCT 24 hours after endovascular stroke therapy did not display signs of infarction or intracranial hemorrhage. On day 2 after endovascular stroke treatment, the patient was transferred to the hematology ward (NIHSS 5, mRS 3). A follow-up MRI was performed on day 10 after endovascular stroke treatment to evaluate the extent of a possible infarction and intracranial hemorrhage as possible contraindications to the continuation of chemotherapy. No hyperintensities on diffusion-weighted imaging (DWI), or changes of fluid attenuated inversion recovery (FLAIR) images as evidence of acute or subacute ischemia were detected (Fig. 1). Time-of-flight MR angiography did not show a renewed occlusion.

In the following days, the patient gradually deteriorated, particularly in terms of apraxia and higher-order language deficit with impaired speech fluidity, comprehension, and repetition. Consequently, the patient was readmitted to the stroke unit on day 23 after the index event (NIHSS 7, mRS 5). Delirium, seizure, or a metabolic cause for these new symptoms were ruled out by means of clinical diagnostics, laboratory tests, and electroencephalogram (EEG). Repeat MRI was performed without signs of diffusion restriction, apparent diffusion coefficient (ADC) pseudonormalization, or a renewed occlusion of the left internal carotid artery. However, new subtle hyperintensities of the white matter, sparing the cortex and U-fibers, were visible on FLAIR images; strictly limited and localized unilaterally to the vascular territory of the left anterior circulation.

The patient’s symptoms gradually improved with best medical treatment (platelet function inhibitor, statin, and antihypertensive drug) as well as physical therapy, occupational therapy, and speech therapy. A detailed list of the medication the patient received in the comprehensive stroke center is listed in Table 2. The patient was transferred from the stroke unit to hematology to continue cancer treatment on day 32 and discharged home on day 40 after endovascular stroke treatment (NIHSS 7, mRS 3).

**Table 2:** List of the medication the patient received during his stay in the comprehensive stroke center

Drug	Dosage	Morning	Noon	Evening	Night	Route of administration	Start	End
Tamsulosin	0.4 mg	0	0	1	0	Orally	Preexisting	Ongoing after 313 FU
Torasemide	10 mg	2	1	0	0	Orally	Preexisting	Ongoing after 313 FU
Eplerenone	25 mg	1	0	0	0	Orally	Preexisting	Ongoing after 313 FU
Esomeprazole	40 mg	1	0	0	0	Orally	Started first day after EST	Ongoing after 313 FU
Acetylsalicylic acid	100 mg	0	1	0	0	Orally	Started first day after EST	Ongoing after 313 FU
Urapidil if BP > 180 mmHg	12.5 mg	As needed	As needed	As needed	As needed	Intravenous	Started on day of EST	Discharge from the stroke unit on day 32 after EST
Metoclopramid in case of nausea up to three times a day	10 mg	As needed	As needed	As needed	As needed	Orally	Started on day of EST	Discharge from the stroke unit on day 32 after EST
Atorvastatin	20 mg	0	0	1	0	Orally	Started first day after EST	Ongoing after 313 FU

*EST*  endovascular stroke treatment, *FU* Follow-up, *BP* Bloodpressure

On day 92 after acute stroke treatment, the clinical and imaging follow-up of the patient was carried out. The patient presented in a significantly improved condition without a walking aid and other support. The patient reported that in everyday life he needs help doing housework, getting groceries, or preparing food, but is not dependent on assistance with banking and legal transactions or maintaining personal hygiene (mRS 2). MRI on day 92 showed that the faint, diffuse hyperintensity in the left anterior circulation territory on FLAIR had not resolved and had not further increased in intensity or spread to other areas of the brain. An additional MRI of the brain was performed 313 days after acute stroke treatment. Compared with the MRI from day 92, there was no change in the imaging findings. With the exception of the restrictions in daily activities already existing on day 92, the patient continues to be self-sufficient.

## Discussion

We report a case of a man in his 80s who showed clinical signs and developed morphological changes on MRI characteristic for DPHL following a dissection-related occlusion of the left carotid artery. In contrast to the usual characteristic bilateral changes on MRI in patients with DPHL, the unique feature of this case report is that it is the first reported case of a clinically primarily cognitive, radiologically strictly unilateral manifestation of DPHL.

DPHL is a rare entity clinically characterized by neurologic deterioration following a period of clinical stability or improvement after an initial episode of hypoxia [3]. DPHL has been associated with respiratory failure in the setting of resuscitation, carbon monoxide intoxication, or in patients with drug overdoses involving different toxins like alcohol, cocaine, heroin, morphine, and benzodiazepines [3–6]. Following hypoxia, extensive hyperintense subcortical white matter changes on T2-weighted and FLAIR images can be identified. White matter changes in DPHL are usually confluent and homogeneous; however, cases with a heterogeneous, patchy appearance have been reported [3]. Of particular note is that T2/FLAIR hyperintensities are bilateral and symmetric and occur under preservation of the U-fibers.

In this case report, the clinical course and characteristic image-morphological changes for DPHL were observed in a patient who was treated for an acute occlusion of the left cervical internal carotid artery. Most importantly, changes on FLAIR hyperintensities were observed strictly limited and localized unilaterally to the left anterior circulation, that is, the vascular territory that was downstream of the initial vascular occlusion, spared U-fibers, not accompanied by a diffusion restriction, and developed over a course of time accompanied by a delayed clinical deterioration of the patient.

## Clinical- and imaging-based differential diagnostics

In patients with global hypoxic-ischemic injury caused by cerebral hypoperfusion in the event of cardiac arrest, drowning, or asphyxiation, injury patterns are highly variable depending on brain maturity, severity, and length of cerebral hypoperfusion [7]. Mild-to-moderate cerebral hypoperfusion might cause watershed zone infarcts, and severe cerebral hypoperfusion might damage gray matter structures including the basal ganglia, thalami, cortex, cerebellum, and hippocampi. Typical for global hypoxic-ischemic injury is increased signal intensity on DWI in cerebellar hemispheres, basal ganglia, and cerebral cortex. In ischemic stroke and global hypoxic-ischemic injury, changes on DWI can be detected very early, sometimes several minutes after arterial occlusion or cerebral hypoperfusion and is primarily due to cytotoxic edema. DWI and ADC abnormalities may pseudonormalize by the end of the first week [8]. ADC pseudonormalization is a normal phase encountered in the subacute stage of ischemic stroke and represents an apparent return to normal healthy brain values on ADC maps, and does not represent true resolution of ischemic damage.

Changes on T2-weighted sequences are usually visible after a few hours in patients with hypoxic brain damage or ischemic stroke. Hyperintensities on FLAIR images can be used to identify acute ischemic strokes at 3 hours or less [9]. In the case of an acute ischemic stroke, hyperintensities intensify in the first 24 hours and are therefore still visible days after the event until scarring or the breakdown of the damaged tissue.

Since in our case the first MRI imaging was done on day 10 after the index event, DWI and ADC changes might not have been captured on MR images. However, hypoattenuation suggestive of ischemic changes were not detected on 24-hour follow-up NCCT. In addition, following acute ischemic stroke or manifest hypoxic brain damage, FLAIR hyperintensities should have already been visible on first MRI imaging on day 10 and not developed later to be visible on MR imaging on day 23.

Other disorders or imaging changes that may resemble DPHL, such as posterior reversible encephalopathy syndrome (PRES), progressive multifocal leukoencephalopathy (PML), or DWI and FLAIR hyperintensities associated with status epilepticus, should be ruled out. In PRES patchy T2/FLAIR hyperintensities sparing the cortex and usually located in parieto-occipital lobes and posterior cortical watershed zones can be observed most often [10]. Atypical PRES as a variant may include the frontal lobes [11]. However, our patient did not have any hypertensive phases and did not show any kind of intracranial hemorrhage on imaging, as it would be observed most often in patients with PRES.

PML is caused by a subacute opportunistic infection caused by DNA virus John Cunningham polyomavirus (JCV) [12]. On T2-weighted images, PML can typically induce hyperintensities, which are predominantly seen in subcortical and periventricular white matter, often involving subcortical U-fibers leading to a scalloped appearance [13]. Since our patient was undergoing treatment for AML, associated PML could have been the trigger of the FLAIR hyperintensities. However, the U-fibers were spared in our patient. In addition, the anatomical distribution of FLAIR hyperintensities is limited to the area downstream of the left internal carotid artery, which in turn makes DPHL more likely and isolated PML in this area less likely.

In status epilepticus, T2/FLAIR hyperintensities of the gray matter and/or subcortical white matter with or without mild mass effect may be observed, and are usually accompanied by acutely restricted diffusion. In some cases, it might be difficult to differentiate clinical symptoms and imaging changes of status epilepticus and ischemic stroke [14]. In our case, clinical course, imaging appearance, and EEG patter of the patient did not match theses differential diagnoses.

Metabolic or toxic disorders, for example, as a neurotoxic side effect of arsenic trioxide and all-trans-retinoic acid, that is, the patients’ chemotherapy for AML, would, if any, have caused more global, symmetrical changes on MR imaging, which is why they are not discussed further in this manuscript, although we have investigated them in clinical care of the patient.

Our publication has some limitations. On the one hand, the first MRI was performed on day 10, which means that no baseline MRI was performed before or 24 hours after acute ischemic stroke therapy. Performing CT initially and on 24-hour follow-up imaging is worldwide standard practice in the diagnostic workup of an acute ischemic stroke; however, as a result, possible early changes on DWI are beyond our knowledge.

Since this is an individual case, our findings cannot be directly transferred or reproduced, and are dependent on colleagues, in particular neurologists and (neuro-)radiologists, to confirm our observations. For this purpose, different distribution patterns and clinical manifestations might be documented according to the affected vascular territories. Therefore, this topic might not be referred to as unilateral DPHL, but territorial DPHL in the future.

## Conclusions

Delayed post-hypoxic injury may present as a unilateral variant limited to a downstream territory following a large vessel occlusion. Delayed clinical symptoms should raise the suspicion of unilateral DPHL after acute ischemic stroke treatment. As radiological signs of unilateral DPHL may be subtle, caution is warranted and repeated controls using MRI might be necessary to correlate and monitor the clinical course and the morphological changes of the brain.

## Data Availability

The data that support the findings of this case report are available from the corresponding author upon reasonable request.

## Abbreviations

ADC: Apparent diffusion coefficient
AML: Acute myeloid leukemia
ASPECTS: Alberta Stroke Program Early CT Score
DPHL: Delayed post-hypoxic leukoencephalopathy
DWI: Diffusion-weighted imaging
FLAIR: Fluid Attenuated Inversion Recovery
mRS: Modified Rankin Scale
NCCT: Non-contrast-enhanced computed tomography
NIHSS: National Institutes of Health Stroke Scale
PRES: Posterior reversible encephalopathy syndrome
